# Neuropathic pain in diabetic patients: a review of pharmacological treatment selection according to psychopathological and somatic factors

**DOI:** 10.3389/fpsyt.2026.1741046

**Published:** 2026-01-26

**Authors:** Artur Reginia, Aleksandra Mazur, Agata Bąba-Kubiś, Marcin Jabłoński, Maciej Sołtysiński, Jerzy Samochowiec, Jolanta Kucharska-Mazur

**Affiliations:** Department of Psychiatry, Pomeranian Medical University, Szczecin, Poland

**Keywords:** comorbidity, depression, diabetes mellitus, neuropathic pain, pharmacological treatment

## Abstract

Diabetes and its complications are becoming an increasingly significant challenge for modern medicine. Diabetes carries a high risk of various complications, including neuropathic pain and certain mental disorders. It is important to note that these two types of complications often interact with each other. Neuropathic pain occurs in up to approximately 50% of patients and is much more common in those with poorly controlled diabetes. A characteristic feature of this type of pain is that it is usually chronic and resistant to conventional analgesics. This limits the available treatment options and necessitates the use of other medications, including certain psychotropic drugs. Among the most common psychiatric disorders are anxiety disorders, which affect about 18% of patients; depressive disorders, present in over 17% of individuals with type 2 diabetes; and sleep disorders, occurring in about 39% of patients. These complications require appropriate and comprehensive management, which cannot always be provided solely by specialists from specific medical fields. Furthermore, it is beneficial to use therapeutic strategies that combine efficacy in alleviating neuropathic pain with an effect on co-occurring psychiatric symptoms. Our search covered the last ten years, was conducted in the databases PubMed and ScienceDirect. The manuscript includes English-language publications, primarily review articles and recommendations that are as up-to-date as possible. The aim of this review is to present the principles of selecting pharmacological treatment for neuropathic pain in patients with diabetes, taking into account coexisting symptoms of depression, anxiety, and sleep disorders.

## 1 Introduction

According to estimates from the latest International Diabetes Federation (IDF) Diabetes Atlas (2025), approximately 11% of the global population suffers from diabetes (1). This disease has a complex pathophysiology, the key feature of which is elevated glucose levels, leading to the development of other metabolic disturbances and the activation of inflammatory processes, ultimately resulting in organ complications. Particular attention should be paid to various forms of microangiopathy and macroangiopathy, as well as the secondary organ damage that arises from them (2). One of the complications is diabetic neuropathy. Although the pathogenesis of this condition is complex and not fully understood, it has been demonstrated that inflammatory reactions, metabolic processes, and microangiopathy are largely involved in the demyelination of nerve fibers. The symptomatology of diabetic neuropathy is diverse and depends on which nerve fibers are affected. It may present as symmetric sensory-motor axonal neuropathy, proximal asymmetric painful motor neuropathy, mononeuropathy, or autonomic neuropathy (3). Symptoms of certain types of diabetic neuropathy include various sensory experiences described as burning, electric shocks, stabbing, hyperalgesia, and allodynia. In addition, symptoms such as numbness, poor balance, and weakness may also be present. Therefore, the sensations described differ from the classical description of pain symptoms (4). The latest analyses suggest that this complication occurs in up to 46% of people with diabetes and indicate risk factors for its development such as female gender, older age, duration of diabetes, and the presence of diabetic nephropathy (5). Another problem is that up to 33% of individuals with type 2 diabetes who reported symptoms of neuropathy did not receive appropriate treatment. Therefore, improvements in the diagnosis and selection of proper therapy for these patients are necessary (6). It is suggested to shift the approach from one focused solely on glycemic control to one that also takes into account multiple metabolic aspects of the disease and weight reduction (7). It is also undeniable that peripheral neuropathy has a negative impact on the quality of life of individuals with diabetes. Moreover compared with those without neuropathy, they scored higher on the anxiety and depression subscales of the HADS questionnaire, which may indicate a deterioration in their mental health (8). Another important point is that individuals with diabetes more frequently experience mental disorders. Among these, the most common by far are sleep disorders, mood disorders, and various anxiety disorders. It has been demonstrated that their prevalence increases with the duration of type 2 diabetes (9, 10). It has also been demonstrated that long-standing diabetes leads to damage to the central nervous system (CNS). Despite certain differences in pathogenesis, CNS damage occurs in both types of diabetes (11). Most studies on depression in people with diabetes do not distinguish organic mood disorders; however, given that diabetes damages the central nervous system, one can infer that the frequency of such diagnoses would also be higher. There is no doubt, though, that the relationship between depression and diabetes is bidirectional (12). The latest American Diabetes Association guidelines recommend annual screening for depression in people with diabetes. Additional screening should be performed after any diabetes-related complication. It is also essential to assess for anxiety disorders and fear of hypoglycemia (13). Although comprehensive guidelines specifically addressing the pharmacological treatment of depression in people with diabetes are lacking, one should draw on the 2009 NICE guideline, which includes recommendations for treating depression comorbid with physical illness, as well as on reports covering particular clinical scenarios and on knowledge of the effects/profiles of individual medications. When the severity of depression warrants pharmacological treatment, selective serotonin reuptake inhibitors (SSRIs) are recommended as the first-line option (14). To date, studies have examined the use of certain SSRIs (sertraline, fluoxetine), TCAs (nortriptyline), and bupropion. Among these, sertraline and fluoxetine were associated with beneficial reductions in glycemia, bupropion appeared promising in this regard, while nortriptyline had an unfavorable effect (15). One of the studies on the effectiveness of venlafaxine compared to fluoxetine in patients after a stroke included some individuals with coexisting diabetes and demonstrated the superiority of venlafaxine (16). Another study conducted by British researchers suggests that the use of SSRIs, TCAs, and mirtazapine is associated with an increased risk of developing diabetes, with a higher risk observed in cases of polytherapy (17). The conducted meta-analysis assessing the effectiveness in terms of depression severity and the impact on HbA1c levels in individuals with diabetes suggested a beneficial effect of agomelatine and escitalopram in both areas studied; however, further research is still needed (18).

The aim of this review is to present the principles of selecting pharmacological treatment for neuropathic pain in patients with diabetes, taking into account coexisting symptoms of depression, anxiety, and sleep disorders.

### 1.1 Depression and diabetes mellitus

Numerous factors influence the likelihood of depression developing in the course of diabetes, including age, gender, education, lifestyle, adherence to therapeutic discipline in diabetes, and abuse of psychoactive substances (19). In addition, diabetes and depression mutually exacerbate each other’s occurrence (12), that can be associated with both the structure and function of the brain, including neurogenesis disorders, as well as oxidative stress, inflammation, glucose and lipid metabolism disorders and the effects of obesity (reduced adiponectin and increased leptin and resistin) (20). Indirect confirmation of this two-way relationship is provided by the fact that metformin use reduces the risk of depression (21).

Meanwhile, as demonstrated in a meta-analysis of 3,898 cases of people with type 2 diabetes, depression and neuropathy are clearly related (OR = 2,01, 95% CI: 1.60–2.54; p < 0.001) (22). This phenomenon can be explained by lower adherence to diabetes treatment recommendations among people with depression and, on the other hand, poorer quality of life among people with neuropathy, which can lead to depression. Inflammatory processes may be a common underlying cause of depression and neuropathy (22).

When selecting antidepressants for diabetes, it is important to assess their metabolic effects. SSRI drugs are not only effective as antidepressants, but are also believed to reduce mortality in metabolic syndrome in diabetes (23). Sertraline did not increase HbA1c. Fluoxetine, on the other hand, improved the body’s homeostasis disturbed by diabetes and increased insulin signaling. On the other hand, SSRIs affect electrical muscle activity and structure properties, thereby increasing the risk of falls and fractures. In addition, they increase the risk of cardiovascular autonomic neuropathy in the type1of DM (20). Studies on the use of SNRIs in the treatment of depression coexisting with diabetes have yielded ambiguous results. Venlafaxine caffeic acid salt has the hypoglycemic effect (24), but on the other hand, the use of antidepressants, including SSRIs, SNRIs, and tricyclic antidepressants increases the risk of type 2 diabetes (using a fixed-effect model (RR, 1.31; 95% CI, 1.26 to 1.37), using random-effect model (RR, 1.49; 95% CI, 1.29 to 1.71) (25), especially at higher doses and with long-term administration (26). However, according to experts from The American Association of Clinical Endocrinology, this is due to the interrelationship between depression and diabetes. The organization’s recommendations emphasize that SSRIs improve glycemic outcomes and contribute to weight loss (27).

In summary, depression and neuropathy are bidirectionally linked – both through common inflammatory and metabolic processes and through their impact on adherence to therapeutic recommendations.

## 2 Methods

Our search covered the last ten years, was conducted between September 2025 and October 2025 in the databases PubMed and ScienceDirect, using ‘neuropathic pain AND diabetes mellitus’ as the main term in combination with other keywords (depression, pharmacological treatment, comorbidity). We found more than 10000 publications, including many doubles, which were deleted in the next step. The final version of the manuscript includes English-language publications, primarily review articles and recommendations that are as up-to-date as possible. When a review articles lacked detailed explanations, empirical sources were also consulted.

## 3 Discussion

### 3.1 Standards for the treatment of neuropathic pain

Painful diabetic neuropathy, a subtype of peripheral painful neuropathy, is specified as “pain as a direct consequence of abnormalities in the peripheral somatosensory system in people with diabetes” (28). Risk factors for painful diabetic neuropathy include, among others: advanced age, increased BMI, longer diabetes duration, and nephropathy (5), each of which complicates treatment. According to French recommendations (29) for the treatment of neuropathic pain, duloxetine and venlafaxine (with a predominance of evidence for the efficacy of duloxetine), TCAs and gabapentin are recommended as first-line treatments for neuropathic pain. Duloxetine is credited with having an analgesic effect independent of its effect on mood. The authors also note the limitations of using these drugs. With regard to TCAs, the authors point out the potential risk of hypotension, urinary retention, exacerbation of glaucoma and cardiac toxicity and recommend using low doses and strict monitoring the patient’s condition at doses higher than 75 mg per day. It is worth emphasizing that the cardiotoxicity of tricyclic antidepressants (TCAs) is primarily manifested by electrocardiographic abnormalities, cardiac arrhythmias, and hypotension. In addition, an increased risk of myocardial infarction has been demonstrated with long-term use of TCAs. Orthostatic hypotension has been reported in up to 50% of patients treated with this class of drugs. These factors should always be taken into consideration when prescribing TCAs to patients with diabetes, who are characterized by an inherently increased cardiovascular risk (30–34). In elderly patients, this is a second-line treatment. Duloxetine, on the other hand, is contraindicated in cases of hepatic or severe renal failure, and gabapentin carries a risk of abuse. The antidepressants carry a low risk of suicidal behavior. Pregabalin is a second-line drug, which may be associated with a potential risk of abuse of this medicine. Although local treatment and an analysis of classical analgesics are not the subject of this paper, it is worth noting that among painkillers, only lidocaine patches are considered first-line treatment, and only for peripheral neuropathy. Cannabinoids, SSRIs and lamotrigine are considered to have controversial efficacy based on available evidence. Given the involvement of serotonergic neurotransmission in pain modulation, attempts have previously been made to evaluate the efficacy of SSRIs in the treatment of neuropathic pain. One of the most recent clinical trials, published in 2008, which assessed the efficacy of escitalopram in patients with painful polyneuropathy, demonstrated a significant reduction in pain symptoms in only a subset of participants, precluding the recommendation of this therapy as standard treatment. In contrast, the results of a randomized, placebo-controlled clinical trial published in 2023, evaluating the efficacy of local lidocaine injections combined with oral citalopram in the treatment of complex regional pain syndrome (CRPS), demonstrated the effectiveness of this therapeutic approach. Overall, the role of SSRIs in the management of neuropathic pain remains inconclusive; therefore, monotherapy with this class of drugs is currently not preferred (35–38). Rosenberger et al. emphasize that three elements should be taken into account in the treatment of diabetic neuropathic pain: control of blood glucose levels, proper care of the limbs, and symptomatic treatment of pain. They give similar recommendations to Moisset et al. regarding pharmacological treatment, except that they place pregabalin on a par with gabapentin. These guidelines summarize the opinions of experts from various working groups, e.g., from the International Association for the Study of Pain (IASP), the European Federation of Neurological Societies (EFNS), the National Institute for Health and Care Excellence of the UK (NICE). The U.S. FDA has approved duloxetine, pregabalin, tapentadol and the 8% capsaicin patch for painful diabetic peripheral neuropathy (39). The American Association of Clinical Endocrinology recommended pregabalin, gabapentin, duloxetine, venlafaxine, amitryptyline, nortryptyline, and capsaicin 8% patch for the treatment of neuropathic pain, or a combination of these medications. The use of opioids is not recommended, and the use of nonsteroidal anti-inflammatory drugs should be avoided due to adverse kidney effects (27) (Blonde). Additional recommendations relate to the patient’s physical condition and do not take into account their mental state. However, no combination therapy has been shown to be superior to monotherapy (40). According to the most recent recommendations of the Neuropathic Pain Special Interest Group (NeuPSIG), α2δ ligands, serotonin–norepinephrine reuptake inhibitors (SNRIs), and tricyclic antidepressants (TCAs) are considered first-line pharmacological treatments for neuropathic pain, with a moderate certainty of evidence and a strong recommendation for first-line use. Based on NeuPSIG guidelines and supported by other scientific evidence, several antidepressants are not recommended for the treatment of neuropathic pain, including SSRIs, mirtazapine, trazodone and bupropion. In addition, according to NeuPSIG recommendations, cannabis-based products are not recommended for use in the treatment of neuropathic pain (41–44).

### 3.2 The treatment of neuropathic pain and mental disorders

The recommendations of The American Association of Clinical Endocrinology suggest regular screening for diabetic polyneuropathy in both type 1 and type 2 diabetes, and strict control of blood glucose levels is recommended as the primary method of prevention. Similarly, screening for depression and insomnia and treatment of these disorders is recommended (27).

According to research by D’Amato et al, polyneuropathy is the greatest risk factor (odds ratio: 4.6, p = 0.038) for depression in diabetes when considering the comorbidity and the various complications of this disease. The same study shows that female gender and pain intensity are predictors of depression severity (45).

Poor quality of night-time sleep, including reduced sleep duration, can lead to daytime sleepiness, reducing physical activity and leading to the development of diabetes complications, including neuropathy, obesity, hypertension, cardiovascular disease and increased mortality (27, 46).

**Figure 1:**
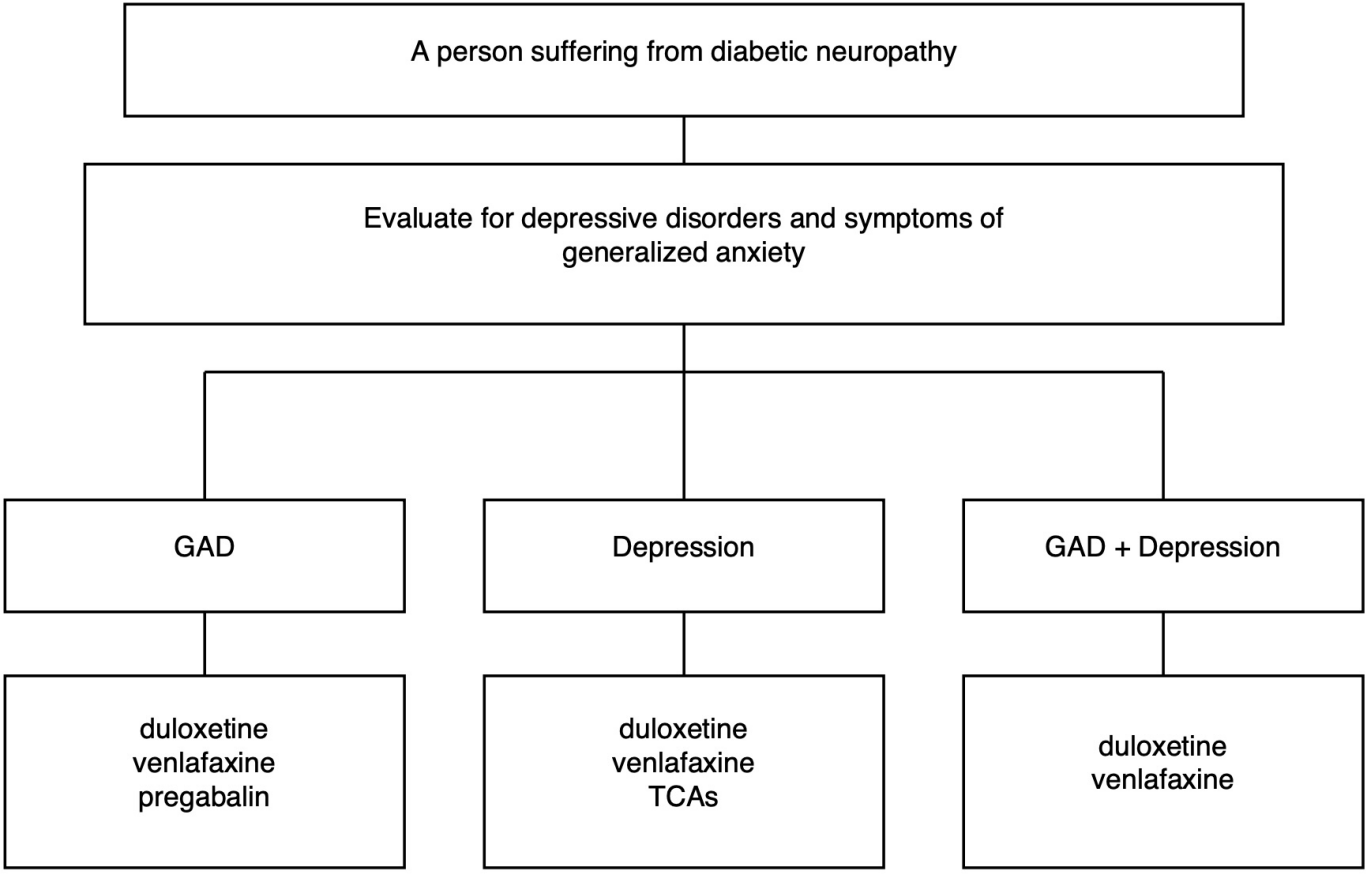
Clinical selection of treatments for neuropathy based on depressive or generalized anxiety symptoms.

There are no clear guidelines in the available literature regarding the treatment of neuropathic pain depending on existing mental disorders. However, after considering various treatment options for this pain, we may consider it appropriate to use NSRI drugs (duloxetine, venlafaxine) and tricyclic antidepressants (amitriptyline, nortriptyline) in cases of coexisting depression. The above drugs and pregabalin can be used in cases of coexisting anxiety disorders. Combining drugs can potentially increase their effectiveness at lower doses (e.g. in the case of combining SNRI/SSRI with pregabalin – these drugs have a synergistic anxiolytic effect). If the patient suffers from insomnia, drugs with a sedative effect (pregabalin, amitriptyline) can be selected. Due to side effects, tricyclic antidepressants should be used in elderly patients in small doses of up to 75 mg per day (47). In general, due to their ambiguous effect on diabetes risk, antidepressants should be used at the lowest effective dose. **Figure 1** illustrates a possible clinical selection of treatments for neuropathy based on depressive or generalized anxiety symptoms.

Controlling normal blood glucose levels and physical exercise are recommended for diabetes, including diabetes with accompanying neuropathy, as a fundamental method of maintaining normal cognitive function. GLP-1 receptor agonists, recommended in diabetic neuropathy, are being studied for the treatment of diabetes in people with reduced cognitive function (48).

## 4 Conclusions

The best results in the treatment of diabetes and its accompanying diseases are achieved by using an integrated treatment model: a combination of pharmacotherapy with complication prevention, psychotherapy, psychoeducation and physical activity.

Diabetic painful neuropathy poses a challenge due to numerous comorbidities, therapeutic difficulties and a significant impact on patients’ daily functioning. Recommended first-line drugs (pregabalin, SNRI, tricyclic antidepressants) are also used in the treatment of comorbid mental disorders. In patients with neuropathic pain and symptoms of depression, dual-action drugs (duloxetine, venlafaxine) are preferred, while in patients with insomnia, amitriptyline or pregabalin are preferred. In patients with neuropathic pain and comorbid generalized anxiety, dual-action agents such as duloxetine and venlafaxine, as well as pregabalin, are preferred. Due to comorbidity in diabetes, the side effect profile of the drug should be carefully analyzed when selecting it and the medicine should be used at the lowest effective dose. As no combination therapy has been shown to be superior, monotherapy should be used instead.

The available data indicate that research is needed on the treatment of diabetic painful neuropathy in specific clinical situations.
